# Histopathological Findings and Metagenomic Analysis of Esophageal Papillary Proliferation Identified in Laying Broiler Breeders

**DOI:** 10.3390/vetsci9070332

**Published:** 2022-06-30

**Authors:** Si-Hyeon Kim, Hye-Soon Song, Chung-Hyun Kim, Yong-Kuk Kwon, Choi-Kyu Park, Hye-Ryoung Kim

**Affiliations:** 1 Avian Disease Division, Animal and Plant Quarantine Agency, 177 Hyeoksin 8-ro, Gimcheon-si 39660, Korea; xdfkm394@gmail.com (S.-H.K.); hssong1217@korea.kr (H.-S.S.); juda1974@korea.kr (C.-H.K.); kwonyk66@korea.kr (Y.-K.K.); 2 College of Veterinary Medicine & Animal Disease Intervention Center, Kyungpook National University, Daegu 41566, Korea

**Keywords:** esophageal papillary proliferation, vitamin A deficiency, metagenomics

## Abstract

**Simple Summary:**

This study describes the histopathological finding of mucosal papillary proliferation observed in the esophagus of chicken and attempts to determine its infectious etiology using metagenomics. Unlike squamous metaplasia of the esophageal mucosa due to vitamin A deficiency, the stratified squamous epithelial cells of the esophagus were completely replaced by increased numbers of ducts/ductules, lymphocytes, and connective tissue, and multiple hyperplasia of the esophageal gland was observed. As the result of the metagenomic analysis of esophagus samples from this chicken, no viral cause was identified; however, the contributing role of *Bradyrhizobium* sp. could not be excluded. In this study, we report the first histopathological examination of a rare case of an esophageal papillary lesion appearing as adenoma in a chicken and highlight the importance of histopathological results for a definitive diagnosis of such cases.

**Abstract:**

White or pale-yellow nodules 2–7 mm in length were observed in the esophageal lumen in a number of laying broiler breeders with reduced laying rates. Metaplasia of the mucosal epithelial layer and mucous gland, as well as lymphocyte infiltration under the esophageal mucous gland and epithelial cell layer, were observed, which we found were caused by vitamin A deficiency. In one chicken, however, the stratified squamous epithelial cells of the esophagus were completely replaced by increased numbers of ducts/ductules, lymphocytes, and connective tissue, resulting in a papillary morphology. The ducts were surrounded by a fibrous stroma. Multiple hyperplasia of the esophageal gland was also observed and the esophageal glands were lined by a single layer of columnar cells, and a large number of lymphocytes were infiltrated into the submucosal layer. Based on the gross findings, this papillary proliferation was considered papilloma, but histopathologically, a mass composed of squamous epithelium was not observed. Therefore, the papillary lesion appeared as adenoma with squamous metaplasia of the esophageal gland and ectasia, or mucosal epithelial papillary hyperplasia, associated with chronic esophagitis. A metagenomic analysis of the esophagus samples from this chicken was performed to determine the infectious etiology. No viral cause was identified; however, a contributing role of *Bradyrhizobium* sp. could not be excluded. In this study, we report the first histopathological examination of a rare case of esophageal papillary proliferation in a chicken and highlight the importance of histopathological results for a definitive diagnosis of such cases.

## 1. Introduction

Vitamin A deficiency causes epithelial damage in the oropharynx and esophagus of chickens. The clinical signs of vitamin A deficiency are initially limited to the proliferation of mucous glands in the esophagus or pharynx. The original epithelium is replaced by a keratinized squamous epithelium through epithelial metaplasia, and the volume increases due to the failure to discharge mucus and necrotic cells. This blocks the mucous glands and results in the appearance of a papilloma or adenoma [1].

Avian papillomatosis is a rare disease in chickens. Cutaneous papillomas are common in psittacine birds [2], and mucosal papillomas of the crop and esophagus, associated with psittacine herpesvirus infection, are most common in pet and aviary birds, such as green-winged or scarlet macaws and Amazon parrots [3]. A previous study reported multiple papillomas in the esophagus and crop of chickens, but did not report the histopathological characteristics [4].

In mammalian species, such as humans, cattle, dogs, and cats, papillomaviruses induce the hyperproliferation of the epithelial cells of the skin or mucosa. Papillomaviruses are a very heterogenous group of viruses and have been implicated in malignant transformation [5]. To date, evidence for papillomavirus-related lesions in poultry has not been reported, although papillomaviruses are thought to cause cutaneous papillomas in pet birds [3]. 

The Avian Disease Division of the Animal and Plant Quarantine Agency (APQA) in South Korea has been conducting comprehensive diagnoses of poultry diseases since 1966. Very few cases of esophageal nodules have been examined, and most of these were found to be histopathologic lesions caused by vitamin A deficiency. Recently, a case of an esophageal nodule showing a specific microscopic lesion was reported [6]. 

Traditional diagnostic methods, such as microorganism culture, polymerase chain reaction (PCR), and serologic assay, require significant time and labor, and are limited methods of determining the etiology of infectious disease [7]. Recent advances in high-throughput sequencing technologies have been used for pathogen detection and discovery [8].

This study presents the histopathological findings of an esophageal papillary proliferation in a chicken and the results of the metagenomic sequencing used to elucidate the causative agent. 

## 2. Materials and Methods

### 2.1. Samples 

Anorexia and a decreased egg-laying rate (from 92% to 60%) were observed in a flock of 27-week-old, well-nurtured broiler breeders. Ten chickens in the flock were submitted to the APQA for disease diagnosis. On the basis of the clinical manifestations and the presence of gross lesions, necropsy, bacteriological culture, and virus isolation using specific-pathogen-free chicken embryonated eggs were performed, following APQA diagnostic protocols. As a control, normal chicken esophagi were collected and tested.

### 2.2. Histopathology 

Sections of organ with distinct lesions were collected and fixed in 5% buffered formalin. The samples were embedded in paraffin blocks and paraffin-wax sections were cut (5 µm), dewaxed, stained with hematoxylin and eosin, and examined by light microscopy. 

### 2.3. PCR Primers and Amplification of Papillomavirus

We performed PCR using papillomavirus primers, BconPVF1 (5-TYCCWAAGGTSTCTGSAAATCA-39) and BconPVR1 (59-CCRAAGCCAATATCKSACAT-39), targeting conserved sequences of the L1 gene of avian, bovine, and human papillomaviruses, as described previously [9]. PCR was performed in a thermal cycler (Eppendorf, Hamburg, Germany) for 1 cycle of 5 min at 95 °C, followed by 95 °C for 1 min, 54 °C for 1 min, and 72 °C for 1 min for 35 cycles, before a final extension at 72 °C for 5 min. 

### 2.4. High-Throughput Sequencing

DNA and RNA were extracted from formalin-fixed paraffin-embedded (FFPE) chicken esophagus samples with histological lesions using the QIAamp DNA FFPE Tissue Kit (Qiagen, Valencia, CA, USA). Low-input DNA was amplified using a random primer (5′-ATCGTCGTCGTAGGCTGCTCNNNNNNNN-3′) as previously described [10]. The PCR program was as follows: initial denaturation at 94 °C for 15 min, 40 cycles of 94 °C for 30 s, 40 °C for 30 s, 50 °C for 30 s, and 72 °C for 1 min, before a final extension at 72 °C for 5 min. cDNA was synthesized using the QuantiTect Reverse Transcription Kit (Qiagen, Valencia, CA, USA). The 16S-23S rRNA gene region of bacteria was amplified using the forward primer 27F (5′-AGAGTTTGATCMTGGCTCAG-3′) and reverse primer 2490R (5′-GACATCGAGGTGCCAAAC-3′), as previously described [11,12,13]. The PCR program was as follows: initial denaturation for 2 min at 94 °C, followed by cycles of denaturation at 94 °C for 30 s, annealing at 58 °C for 30 s, and extension at 72 °C for 1 min. The final extension was for 5 min, at 72 °C. Amplified products were cleaned up using Agencourt AMPure XP (Beckman Coulter, Brea, CA, USA). Viral and bacterial metagenomic libraries were prepared separately using the SQK-LSK109 kit (Oxford Nanopore Technologies, Oxford, UK) and sequenced separately using Minion (Oxford Nanopore Technologies, Oxford, UK).

### 2.5. Bioinformatic Analysis

Trimming was performed using fastp (ver.0.20.0) [14]. Reads aligned to the chicken host genome (accession no. GCF_016699485.2 and GCA_000002315.5) were removed using BWA (ver.0.7.17) and samtools (ver.1.13) [15,16]. Metagenomic sequences were annotated using Kraken2 (ver.2.1.2) with the non-redundant nucleotide (nt) database [17,18]. Visualization of the metagenomic community using the Krona (ver.2.8.1) [19]. The 16S-23S rRNA gene region of bacterial reads was selected with a size range of 2000–3000 bp, after which chimera reads were removed using the VSEACH (ver.2.17.1) [20]. To annotate the bacterial reads, homology-based (BLAST) classification (ver.2.12.0) based on the nucleotide sequence was performed using the in-house bacterial 16S-23S database. Taxonomy was determined using in-house script based on the NCBI taxonomy database (blastn e-value cutoff ≤ 1 × 10^−10^) [21].

## 3. Results

### 3.1. Gross Findings and Histopathology

A number of white or pale-yellow nodules 2–7 mm in length were observed in the lumen of the esophagus. These nodules were found in six out of ten individuals. Most of the nodules were oval and grain-like in shape, but some were round or irregular.

**Figure 1:**
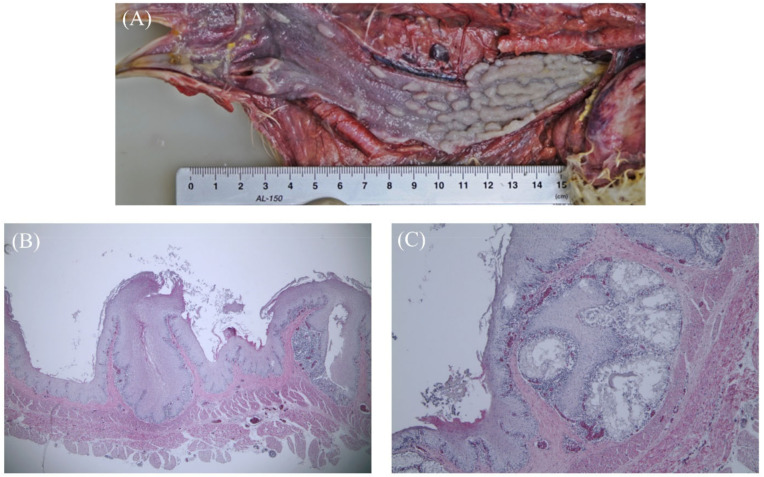
Squamous metaplasia (vitamin A deficiency) in 27-week-old chickens. (**A**) Distended, impacted mucosal-gland-like pustules in the esophagus. (**B**) Squamous metaplasia has replaced all and distention has resulted from occlusion of opening and accumulation of keratin and cellular debris in the lumen (40x). (**C**) Lymphocyte infiltration and hemorrhage in the esophageal gland (40x).

Figure 1 shows squamous metaplasia caused by vitamin A deficiency. Grossly distended, impacted mucosal glands were seen in the lumen. Histopathologically, squamous metaplasia replaced all the epithelium, and distention resulting from luminal occlusion, as well as the accumulation of keratin and cellular debris in the lumen, were observed. Compared to normal esophageal tissue, metaplasia of the mucosal epithelial layer and mucous gland occurred, resulting in keratinization, proliferation, and the elevation of epithelial cells. Lymphocyte infiltration under the esophageal mucous gland and epithelial cell layer was also observed in five chickens. Mucosal papillary proliferation in the esophagus was found in only one chicken. The gross examination revealed irregular, pleomorphic mucosal glands that resembled pustules in the lumen of the esophagus. 

**Figure 2:**
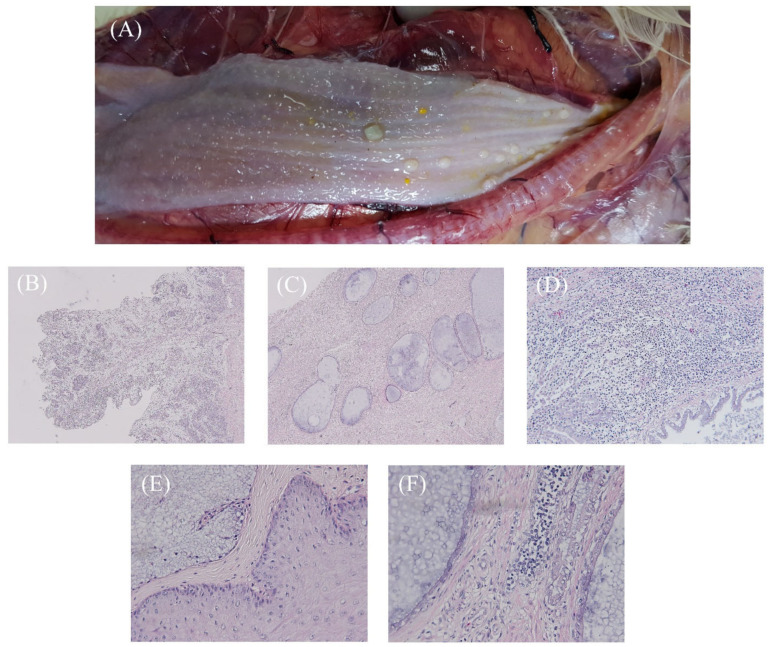
Mucosal papillary proliferation in 27-week-old chickens. (**A**) Irregular, pleomorphic mucosal-gland-like pustules in the esophagus. (**B**) The stratified squamous epithelial cells of the esophagus were completely replaced by increased numbers of ducts/ductules, lymphocytes, and connective tissue, resulting in a papillary morphology (40x). (**C**) Multiple hyperplasia of esophageal gland in the submucosa (40x). (**D**) Degeneration of the epithelial layer, lymphocyte infiltration of the submucosal layer and the ducts surrounded by a fibrous stroma (200x). (**E**) Normal esophageal epithelial cells and esophageal glands (400x). (**F**) The esophageal glands were lined by a single layer of columnar cells, and a large number of lymphocytes infiltrated into the submucosal layer (400x).

Histopathologically, the stratified squamous epithelial cells of the esophagus were completely replaced by increased numbers of ducts/ductules, lymphocytes, and connective tissue, resulting in a papillary morphology. The ducts were surrounded by a fibrous stroma. Multiple hyperplasia of the esophageal gland was also observed and the esophageal glands were lined by a single layer of the columnar cell, and a large number of lymphocytes were infiltrated into the submucosal layer (Figure 2). In addition, we observed visceral gout, liver hemorrhage, and airsacculitis, as well as cytoplasmic dilation and hyperemia between hepatocytes, focal necrosis, and inflammatory cell infiltration around the portal vein in the liver.

### 3.2. Metagenomics Analysis

**Figure 3:**
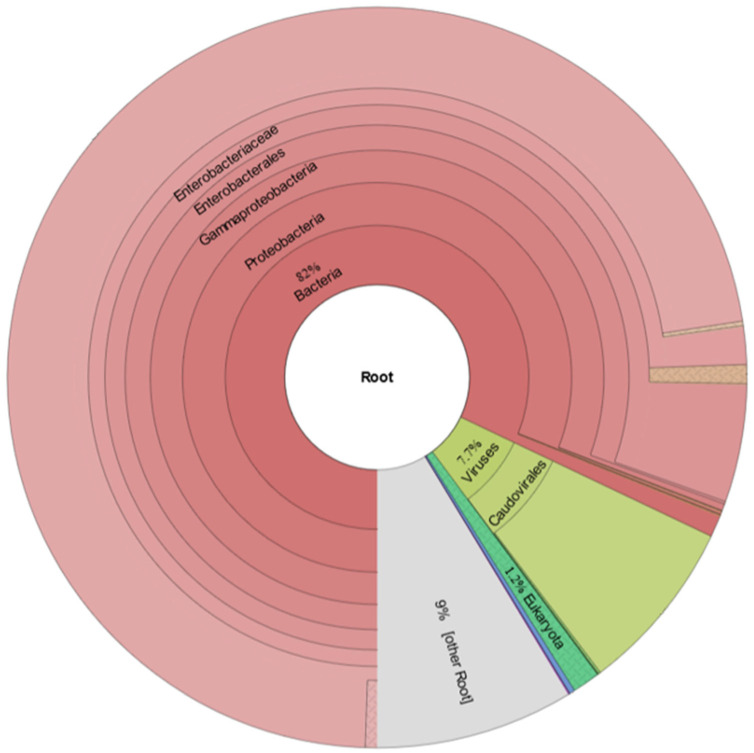
Summary of metagenomics of esophageal papillary proliferation sample used in the study.

To identify the etiology associated with the esophageal papillary proliferation in the broiler breeders, an esophageal sample was sequenced, yielding 8,139,652 reads, 7028 of which were unmapped reads. The N50 value was 3555, and 6548 of the unmapped reads were longer than 1000 bp. The bacterial reads accounted for 82% of the unmapped reads, and most of the bacterial reads were classified as *Escherichia coli.* The viral reads accounted for 7.7% of the unmapped reads, and most of the viral reads were classified as bacteriophage (Figure 3). 

To identify the bacteria, the bacterial 16S-23S rRNA gene region was amplified and sequenced. A total of 56,412 bacterial reads were obtained from the esophageal papillary proliferation sample, with a dominance of *Bradyrhizobium* sp. (85.1%), *Sphingomonas* sp. (8.1%), and *Gordonia* sp. (6%) (Table 1). A total of 80,013 bacterial reads were obtained in the control sample, with a dominance of Lactobacillaceae (49.2%), *Staphylococcus* sp. (11.6%), *Veillonella* sp. (8%), and *Bifidobacterium* sp. (7.4%). Most of the reads in the esophageal papillary proliferation sample were *Bradyrhizobium* sp., which was not found in the control (Table 1).

### 3.3. Definitive Diagnosis

This flock was diagnosed with multiple diseases. *E. coli* was cultured and identified from the liver samples. Chicken infectious bronchitis virus was detected from the kidney and cecal tonsil samples by RT-PCR, although it was not cultured using embryonated eggs. A comprehensive review, including history, gross findings, and histopathology diagnosed the broiler breeder flock with vitamin A deficiency, papillary proliferation, and colibacillosis. The presence of papillomavirus was investigated by PCR, but no papillomavirus was detected.

## 4. Discussion

Mucosal papillary proliferation were observed in a chicken, with proliferative masses projecting into the lumen of the esophagus. The lesions closely resembled those caused by papillomaviruses in mammals, but no papillomavirus was detected by PCR. Based on the gross findings, this papillary proliferation was considered papilloma, but histopathologically, a mass composed of squamous epithelium was not observed. The stratified squamous epithelial cells of the esophagus were completely replaced by increased numbers of ducts/ductules, lymphocytes, and connective tissue, resulting in a papillary morphology. The ducts were surrounded by a fibrous stroma. Multiple hyperplasia of the esophageal gland was also observed, the esophageal glands were lined by a single layer of columnar cells, and a large number of lymphocytes were infiltrated into the submucosal layer. 

The viral metagenomics analysis used to identify the infectious etiology of the esophageal sample revealed only bacteriophages. Bacterial metagenomics using the 16S-23S rRNA gene region identified *Bradyrhizobium* sp. in the esophageal papillary proliferation. In humans, *Bradyrhizobium enterica* is associated with mucosal erythema, paneth-cell metaplasia, colitis, and granuloma in the upper and lower gastrointestinal tract [22,23,24]. Currently, it is not known whether *Bradyrhizobium* sp. causes similar disease in chickens. *Bradyrhizobium* sp. is considered a normal flora in the chicken intestine, yet it accounted for most of the bacteria identified in the esophagus of the chicken with papillary proliferation in this study. It is unclear whether this was an opportunistic infection by *Bradyrhizobium* sp. after papillary proliferation formation, or whether *Bradyrhizobium* sp. caused the esophageal papillary proliferation.

Typical neoplasms in chickens are induced by viruses, such as Marek’s disease virus, avian leucosis virus, and reticuloendotheliosis virus. Other neoplastic diseases in the gastrointestinal systems of birds include squamous-cell carcinoma (SCC), papilloma, adenomas, and adenocarcinomas. SCC has been reported in the esophagus, larynx, and follicles, and is thought to be caused by feeding and water management; however, the exact cause is unknown [25,26,27,28,29,30,31]. In SCC, macroscopically observed mucosal lumps are accompanied by necrosis and bleeding, and histological findings show that keratinocytes form in the center of the epithelial tissue, and island-shaped lesions surrounded by squamous epithelial cells appear. This is different from the papilloma lesions observed in the chicken in this study. Cases of papillomas in chickens are very rare, and although histological characteristics have not previously been reported, they may occur in the esophagus and follicles. In birds such as parrots, papillomas are characterized by the proliferation of mucosal epithelial cells and lymphocyte infiltration around the connective tissue. In these cases, the cause is known to be related to herpes-virus infection [3,4,31]. No viral cause was identified in the chicken in this study, and histopathologically, the papillary lesion appeared as fibroadenoma. Proventricular adenomas and adenocarcinomas of the small intestine in chickens have been described [3], but there were no reports of esophageal adenoma. The histopathological lesions observed in this study were neither SCC nor papilloma, but were considered adenoma made up of esophageal-gland squamous metaplasia and ectasia with severe chronic esophagitis. Therefore, this study offers a meaningful histological description of a lesion presumed to be esophageal adenoma.

Vitamin A is a fat-soluble vitamin, similar to vitamin D and vitamin E. In general, corn-based feed is rich in beta-carotene, and since beta-carotene is a precursor of vitamin A, severe deficiency does not occur in most chickens [32]. However, it is an essential nutrient, and its additional administration to young chicks during growth or hens that have reached their laying peak is required. In this study, the chickens with esophageal nodules were broiler breeders that had reached their laying peak and were diagnosed with vitamin A deficiency. After supplying nutritional supplements after the disease diagnosis, the egg-laying rate returned to normal. The appearance of esophageal nodules is an early sign of vitamin A deficiency and can often occur in laying breeders. However, if clinical signs become severe, the damage can be amplified by secondary infection with viruses and bacteria [33]. Based on these observations, esophageal papillary proliferation in chickens can be said to be mucosal epithelial papillary hyperplasia associated with chronic esophagitis induced by the effects of vitamin A deficiency or unknown factors related to the environment.

In this study, we reported the first histopathological assessment of a rare case of esophageal papillary proliferation in a chicken and highlighted the importance of histopathological examination for obtaining a definitive diagnosis of such cases. The esophageal papillary proliferation in this chicken was idiopathic, and no viral cause was identified; however, a contributing role of *Bradyrhizobium* sp. could not be excluded. Further case studies are needed.

## Figures and Tables

**Table 1:** Bacteria identified using the 16S-23S rRNA gene region with esophageal papillary proliferation sample and control.

Bacteria	Reads	
	Esophageal Papillary Proliferation (% Total Reads)	Control (% Total Reads)
*Bradyrhizobium* sp.	48,023 (85.1)	-
*Sphingomonas* sp.	4565 (8.1)	-
*Gordonia* sp.	3400 (6)	-
*Ralstonia pickettii*	261 (0.5)	-
*Clostridium beijerinckii*	3 (<0.1)	-
Lactobacillaceae	2 (<0.1)	39,355 (49.2)
*Escherichia coli*	2 (<0.1)	302 (0.4)
*Staphylococcus* sp.	-	9303 (11.6)
*Veillonella* sp.	-	6423 (8)
*Bifidobacterium* sp.	-	5927 (7.4)
*Corynebacterium* sp.	-	1693 (2.1)
*Kurthia* sp.	-	1659 (2.1)
*Gallibacterium anatis*	-	1409 (1.8)
*Rothia* sp.	-	152 (0.2)
*Enterococcus*	-	12 (<0.1)
Uncultured bacterium	156 (0.3)	13,778 (17.2)
Total	56,412	80,013

## Data Availability

The data that support the findings of this study are available from the corresponding author upon reasonable request.

## Abbreviations

APQA	Animal and Plant Quarantine Agency
nt	Non-redundant nucleotide
PCR	Polymerase Chain Reaction
SCC	Squamous Cell Carcinoma
